# Contractions from Rheumatism and Old Cases of Anchylosis of the Knee-joint Cured by Chloroform

**Published:** 1855-10

**Authors:** 


*Contractions from Rheumatism and old cases of Anchylosis of the Knee-joint cured by Chloroform.* (Under the care of Mr. Erichsen.)—Five cases of contraction of limbs from rheumatism and from old anchylosis of the knee-joint and other joints, under care during the last few months at University College Hospital, requiring different plans of of practice, but illustrating well the resources opened up by chloroform treatment, have appeared to us very deserving of notice in a practical point of view.

These cases, under treatment at different times, have been the result of chronic inflammatory disease of the knee-joint; in all of them the leg was bent on the thigh to different degrees, varying from a right to a more or less acute angle; in none of the cases could the patients put the foot to the ground or use it in progression. The plan of treatment adopted by Mr. Erichsen is, to place the patient fully under the effects of chloroform, so as to relax the muscles of the limb, and render the patient insensible to the pain of the operation; then, as the lesser evil of the two, forcibly to straighten or extend the bent-up leg. In doing this, we noticed on more than one occasion loud snaps or cracks in the joint —not a little alarming at first, though without danger—as the false membranes within and ligamentous structures outside the joint were broken through. The extended limb is then put up in a straight splint, evaporating ether lotions applied, and the patient kept quiet. Generally this plan suffices to straighten the limb, but in some very obstinate instances division of the hamstring tendons is required; this, however, Mr. Erichsen finds it rarely necessary to employ, only one of the five following cases requiring it.

CASE 1.—A young woman, aged twenty-two, who had been attacked, April, 1855, with acute rheumatism and rheumatic contraction of the left knee, the latter bent at nearly a right angle and excessively painful, came under Mr. Erichsen's care, who succeeded in straightening the limb under chloroform. The subsequent inflammation was easily reduced by leeches and slight mercurials. She appears latterly quite well, and wears the starch bandage. She would not allow the limb even to be touched previous to the use of the chloroform.

CASE 2.—A man, aged twenty-three, came under care in July, with contraction of his left knee, which was bent at right angles, and had been in this condition for eight months, the result of inflammation of the joint. There was no pain or uneasiness about it; very limited motion, not more than about two inches when he attempted to move the foot. Mr. Erichsen had him placed under the influence of chloroform, and then forcibly stretched the limb; loud crackling noises were heard as if something were being crunched or torn through. There were no inflammatory symptoms subsequently; and he was able to leave the hospital in ten days, merely wearing starch bandages.

CASE 3.—A woman, aged thirty; anchylosis nearly complete of left knee for nine months, consequent on rheumatism; scarcely any motion in the joint; no pain or tenderness. The limb here also was straightened, or forcibly extended, under the influence of chloroform. The extension was attended with loud snapping or crackling of the old adhesions. Some severe inflammatory action followed, which, however, was easily subdued by evaporating lotions. This woman also had starch bandages applied; and left the hospital quite improved in health.

CASE 4.—A woman, aged forty-two, whom we saw operated on early last month. She complained of anchylosis of the knee, with bent or twisted condition of the fore-arm and hand, all the result of rheumatic inflammation contracted two years since. Mr. Erichsen straightened the limbs as usual under chloroform; though at first he believed that he should have had recourse to division of the tendons. Loud crunching sounds were heard, as in the former cases. This case also has done well.

CASE 5.—This was an instance of anchylosis of the left knee, in a female, aged thirty-two, of not less than sixteen years' standing, during the whole of which period the limb had not been put to the ground. The leg was considerably wasted, though nearly as long as the healthy limb; it was bent nearly at a right angle; the hamstring tendons were very tense. Mr. Erichsen having placed the patient under chloroform, divided these tendons, and then forcibly straightened the limb, tearing down apparently to us, several old adhesions, in and around the joint. No inflammatory action supervened; and this limb is now in a straight position, supported by starch and gum bandages.

These cases may not claim any superiority over many others of a like kind by their originality, yet they are deserving of notice for their practical value.—*The Lancet.*

